# Supplementary material for: MetaRibo-Seq measures translation in microbiomes
Source: Nat Commun. 2020 Jun 29;11:3268. doi: 10.1038/s41467-020-17081-z (PMC7324362; doi:10.1038/s41467-020-17081-z)
Supplement: Supplementary file 10 — Supplementary Data 7 [file 41467_2020_17081_MOESM10_ESM.zip › File2/Confidence_High_Taxonomy/53858_out.krona.html]

Javascript must be enabled to view this page.

members
magnitude
magnitudeUnassigned
count
unassigned
taxon
rank

53858\_out

2

2
superkingdom
2

2
976
phylum

class
200643
2

2
171549
order

family
815
2

816
genus
2

species

SRS149181\_contig\_number\_contig-100\_3583.123689SRS893256\_contig\_number\_2896
820
2
